# Physical Activity Attenuates the Obesity-Induced Dysregulated Expression of Brown Adipokines in Murine Interscapular Brown Adipose Tissue

**DOI:** 10.3390/ijms221910391

**Published:** 2021-09-27

**Authors:** Takuya Sakurai, Toshiyuki Fukutomi, Sachiko Yamamoto, Eriko Nozaki, Takako Kizaki

**Affiliations:** 1 Department of Molecular Predictive Medicine and Sport Science, Kyorin University School of Medicine, 6-20-2 Shinkawa, Mitaka, Tokyo 181-8611, Japan; kizaki@ks.kyorin-u.ac.jp; 2 Department of Pharmacology and Toxicology, Kyorin University School of Medicine, 6-20-2 Shinkawa, Mitaka, Tokyo 181-8611, Japan; fukutomi@ks.kyorin-u.ac.jp; 3 Department of Chemistry, Kyorin University School of Medicine, 6-20-2 Shinkawa, Mitaka, Tokyo 181-8611, Japan; s_yamamoto@ks.kyorin-u.ac.jp; 4 Core Laboratory for Proteomics and Genomics, Kyorin University School of Medicine, 6-20-2 Shinkawa, Mitaka, Tokyo 181-8611, Japan; enozaki@ks.kyorin-u.ac.jp

**Keywords:** brown adipose tissue, brown adipokine, obesity, physical activity, brown adipocyte differentiation

## Abstract

In recent years, brown adipose tissue (BAT), which has a high heat-producing capacity, has been confirmed to exist even in adults, and it has become a focal point for the prevention and the improvement of obesity and lifestyle-related diseases. However, the influences of obesity and physical activity (PA) on the fluid factors secreted from BAT (brown adipokines) are not well understood. In this study, therefore, we focused on brown adipokines and investigated the effects of obesity and PA. The abnormal expressions of gene fluid factors such as galectin-3 (*Lgals3*) and Lgals3 binding protein (*Lgals3bp*), whose proteins are secreted from HB2 brown adipocytes, were observed in the interscapular BAT of obese mice fed a high-fat diet for 4 months. PA attenuated the abnormalities in the expressions of these genes. Furthermore, although the gene expressions of factors related to brown adipocyte differentiation such as peroxisome proliferator-activated receptor gamma coactivator 1-α were also down-regulated in the BAT of the obese mice, PA suppressed the down-regulation of these factors. On the other hand, lipogenesis was increased more in HB2 cells overexpressing Lgals3 compared with that in control cells, and the overexpression of Lgals3bp decreased the mitochondrial mass. These results indicate that PA attenuates the obesity-induced dysregulated expression of brown adipokines and suggests that Lgals3 and Lgals3bp are involved in brown adipocyte differentiation.

## 1. Introduction

With the global increase in the number of lifestyle-related diseases such as type 2 diabetes, patients with obesity caused by the hypertrophy of white adipose tissue (WAT) have become a global issue [1,2]. In 2016, the World Health Organization (WHO) announced that the number of diabetes patients reached 422 million in 2014, which is almost four times higher than the case count (108 million) in 1980 [3]. Direct fatal cases of diabetes increased to 1.5 million in 2012, and higher blood glucose caused an additional 2.2 million deaths by increasing the risks of cardiovascular and other diseases [3]. Additionally, it is estimated that 43% of all deaths due to high levels of blood glucose occur before the age of 70, and the population of diabetes patients is expected to exceed 700 million by 2045 if no efficient preventative strategies can be enacted [3,4]. Hence, it is crucial to establish efficient preventative and improvement strategies for obesity and lifestyle diseases. Exercise is, in fact, widely accepted as an efficient way to prevent and improve the issues of obesity and lifestyle diseases [5,6]. For example, Helmrich et al. [7] tracked 5990 male university graduates and found that, for each 500 kcal increase in energy expenditure during exercise per week, the risk of developing type 2 diabetes was decreased by 6%. Another study of 21,271 male physicians in the United States over 5 years demonstrated that members of the group who exercised to the point of breaking into a sweat once per week had a lower risk of developing diabetes [8].

There are two types of adipose tissue. The first is WAT, which stores excess energy as triglycerides and contributes to energy supply when needed, such as during physical activity (PA) and aerobic exercise. The second is brown adipose tissue (BAT), which converts energy into heat and contributes to the maintenance of body temperature. WAT produces a wide variety of humoral factors generally called “adipokines”, such as adiponectin, tumor necrosis factor-α, and C-C motif chemokine 2 (Ccl2), which is also known as monocyte chemoattractant protein-1. A number of studies showed that insulin resistance is one of the main pathophysiological features of type 2 diabetes, and this condition is induced in skeletal muscle, the liver, and white adipocyte by WAT inflammatory changes due to the dysregulated expression of adipokines in hypertrophic WAT [9,10,11]. Thus, this condition has attracted much attention as the cause of lifestyle-related diseases, and multiple reports listed it as a target for the effect of PA and exercise [12,13].

Meanwhile, BAT is activated by noradrenaline being released during sympathetic nervous activities such as exposure to coldness [14,15]. In activated BAT, energy is converted to heat by the abundant effect of uncoupling protein 1 (UCP1) in mitochondrion [14,15]. BAT is thought to abundantly exist exclusively in small animals such as mice and in human infants, but it almost totally disappears in adults and, as such, has been assumed to carry no biological significance. However, improvements in inspection technologies recently confirmed a sufficient amount of BAT in adults, and, moreover, the degree of obesity was inversely correlated with the level of BAT activation [16,17,18]. Cold-induced, whole-body energy expenditure in humans with BAT is substantially increased compared with those without BAT, and reports documented that continuous cold stimulation reduces obesity. Therefore, BAT has become a target for the improvement of obesity [19,20,21]. Furthermore, both cold stimulation and stimulation with drugs were shown to have the same effect as noradrenaline by inducing adipocytes similar to brown adipocytes in murine subcutaneous WAT [22,23]. These cells are called beige cells, or bright cells, and are different from classic conventional brown adipocytes [22,23]. Since browning was observed in rat subcutaneous WAT even after running exercise, this phenomenon is now under active investigation [24].

In addition to WAT being reported as secreting various adipokines, humoral factors from BAT, which are called brown adipokines, or batokines, are now being reported [25,26,27]. For example, studies are showing that fibroblast growth factor 21 is secreted by BAT and acts on WAT to improve glucose and lipid metabolism, and this promotes WAT browning through increased expression of peroxisome proliferator-activated receptor γ coactivator-1α (PGC-1α) [26]. Neuregulin 4 is also secreted by BAT and is reported to be involved in lipid biosynthesis in the liver [26]. The recently identified novel brown adipokine ependymin-related 1 was suggested to be important for brown fat commitment [28]. Nevertheless, little is known about the effects of obesity and PA on brown adipokines. In the present study, therefore, we identified humoral factors from HB2 brown adipocytes [29] and investigated the effects of obesity and PA on brown adipokines in mice interscapular BAT.

## 2. Results

### 2.1. Identification of Humoral Factors Secreted from HB2 Brown Adipocytes

Exhaustive analysis on humoral factors secreted from brown adipocytes was performed by various research groups in recent years [28,30,31]. We used mass spectrometry in an attempt to identify humoral factors secreted from mouse HB2 cells fully differentiated in brown adipocyte. Complement C3 and fatty acid binding protein 4 (Fabp4) protein showed high values for peptide-spectrum matches (PSMs) in the culture medium of HB2 brown adipocytes (Table 1). Furthermore, humoral factors such as transferrin, apolipoprotein E (ApoE), EGF-containing fibulin-like extracellular matrix protein 1 (Efemp1), complement factor D (Adipsin), Ccl9, galectin-1 (Lgals1), Lgals3, and Lgals3 binding protein (Lgals3bp) were confirmed in the culture medium of HB2 brown adipocytes (Table 1). Extracellular matrix proteins collagen (Col) 6a1, Col6a2, Col1a2, and fibronectin were found as well (Table 1).

### 2.2. Physical Characteristics of Animals

Physical activity is defined as any bodily movement produced by skeletal muscles that results in energy expenditure above resting levels [32]. We selected voluntary wheel running as a method of PA. Compared to forced running exercise on a treadmill, voluntary wheel running has the advantage that it can be performed under non-stress conditions and is similar to the natural running behavior of mice [33]. Voluntary wheel running has various beneficial effects in mice, such as promoting mitochondrial biogenesis in skeletal muscle, and attenuates dysregulated expression of adipokine genes in WAT due to obesity [33,34].

Final body masses (g, mean ± SE, *n* = 4 in each) were significantly higher in high-fat diet (HFD)-induced obese mice (HFD mice) (37.6 ± 1.6) and HFD mice subjected to voluntary PA (HFD + PA mice) (33.8 ± 0.8) than in control mice (26.9 ± 0.8) at the 4 month time point of HFD intake and PA (Table 2). The final percent of epididymal WAT mass per body mass (%, mean ± SE, *n* = 4 in each) was also significantly higher in HFD mice (5.6 ± 0.3) than in control mice (1.2 ± 0.1). The final percent of epididymal WAT mass per body mass of HFD + PA mice (4.4 ± 0.1) was also significantly higher compared with that of control mice but significantly lower than that of HFD mice (Table 2). On the other hand, no significant differences were observed in the final percent of interscapular BAT mass per body mass among control (0.24 ± 0.01), HFD (0.29 ± 0.05), and HFD + PA mice (0.23 ± 0.02) (Table 2).

### 2.3. Effects of Obesity and Physical Activity on the mRNA Levels of Humoral Factors in the Interscapular BAT of Mice

In consideration of the protein in the HB2 brown adipocytes secretion shown in Table 1, we next used DNA array analysis to investigate the effects of HFD-induced obesity and PA on the expression of genes for humoral factors in the interscapular BAT of mice (Table 3). In the interscapular BAT with obesity due to the intake of an HFD, the expressions of genes for *Lgals3*, *Lgals3bp*, *Ccl2*, and *Ccl9* were markedly upregulated compared with those of the control group. PA significantly suppressed the obesity-induced increases in *Lgals3*, *Lgals3bp*, and *Ccl2* genes in BAT and tended to decrease the enhanced expression of *Ccl9* genes in the BAT of HFD mice. Additionally, expression of the gene for matrix metalloproteinase 12 (*MMP12*) was found in brown adipose tissue. These expressions were increased with obesity, but PA significantly suppressed the obesity-induced increase. However, the secretion of MMP12 protein from HB2 cells could not be confirmed by analysis using mass spectrometry. On the other hand, HFD-induced obesity significantly reduced the expressions of genes for *complement factor C3*, *transferrin*, and *resistin* in the BAT compared with that of control mice, and PA had no effect on the expression of these genes. Moreover, regarding the *adipsin* and the *ApoE* in BAT, PA further augmented the inhibitory effects on gene expression caused by obesity. In addition, the expression of *Efemp1* gene was low only in the BAT of the group with a combination of obesity and PA. No significant differences were observed in the expressions of genes for other humoral factors, such as *Fabp4* and *prosaponin*, in the BAT among control, HFD, and HFD + PA mice. On the other hand, when compared with the control mice, the expressions of genes for some extracellular matrix proteins *Col3a1* and *Col6a1*, along with *fibronectin*, were significantly upregulated in BAT associated with obesity, and these upregulations were attenuated by PA.

### 2.4. Effects of Obesity and Physical Activity on the mRNA Levels of Macrophage Markers and Brown Adipocyte Differentiation-Related Factors in the Interscapular BAT of Mice

The BAT of obese mice displayed increases in the macrophage marker genes such as *F4/80*, *CD53*, and *68* expression by comparison with the control group, but there were no significant differences between the levels in the control and the HFD + PA groups (Table 4 upper). Moreover, although the significantly increased expressions of genes for macrophage-expressed gene 1 (*Mpeg1*) and *CD11b* in the BAT of HFD mice were observed, PA significantly inhibited the obesity-induced increases in the expressions of these genes (Table 4, upper).

Next, we noted the expression changes in the genes for brown adipocyte markers in the interscapular BAT of each group from the DNA array results (Table 4 lower). Although expression of the *UCP1* gene in BAT did not differ among the three groups, the expression levels of the *PGC1-α* gene in HFD mice were significantly lower than those in control mice. The PGC1-αis a master transcription factor coactivator that regulates the expressions of many genes involved in energy production and heat expenditure [35]. This obesity-induced decrease in *PGC1-α* gene expression was not observed in HFD + PA mice. Furthermore, the expressions of genes for type II iodothyronine deiodinase (*Dio2*), which encodes a thyroid hormone-converting enzyme [36], and for lipoprotein lipase (*LPL*), which encodes a lipolytic enzyme involved in catalyzing the hydrolysis of triglycerides in chylomicrons and very low-density lipoprotein particles [37], were significantly declined in HFD and HFD + PA mice compared with that in control mice. The expression level of the very long-chain fatty acid protein 3 (*Elovl3*) gene, which encodes the extremely long-chain fatty acid elongase [38], was lower in HFD mice than in control mice, but there was no statistical difference between the control and the HFD + PA mice. The CCAAT/enhancer binding protein α (C/EBPα) is a critical transcription factor for brown adipocyte differentiation [39]. Expression of the gene for *C/EBPα* in BAT was affected by neither HFD intake nor PA. On the other hand, the *PR domain containing 16* genes, which stimulates brown adipocyte differentiation from progenitor cells [40], was low in the BAT of each group, and no differences were found (data not shown).

### 2.5. Establishment of Brown Adipocytes Overexpressing Lgals3 and Lgals3bp

**Figure 1:**
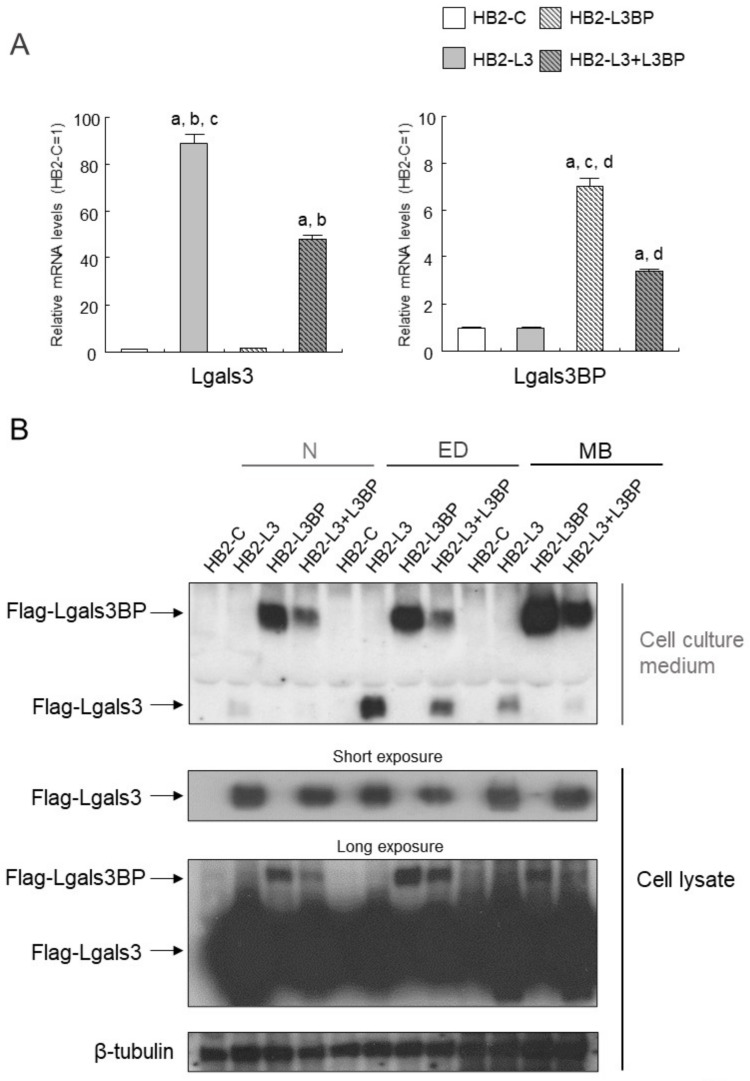

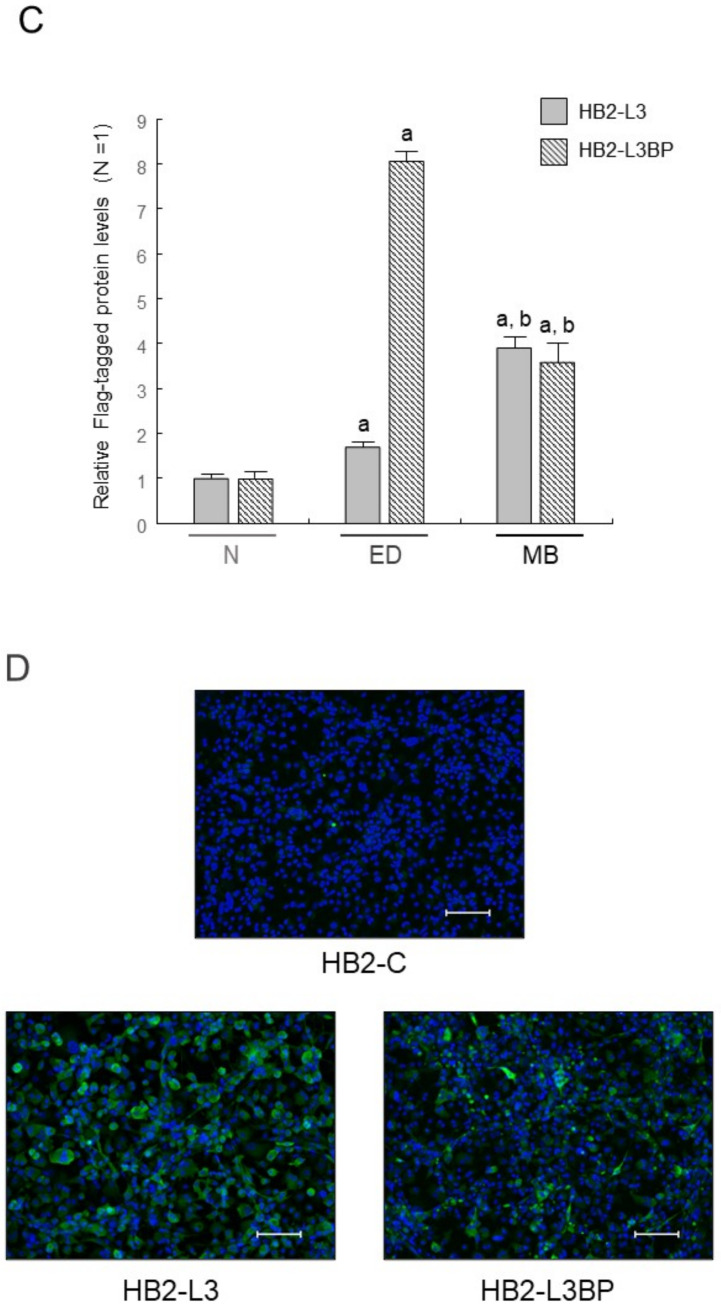
Establishing HB2 cells that overexpress Lgals3 and Lgals3bp. (**A**) Expression levels of *Lgals3* and *Lgals3bp* mRNA in HB2 transfectants. Total RNAs were extracted from each mature HB2 transfectant and then subjected to real time quantitative PCR. The expression levels of *Lgals3* or *Lgals3bp* mRNA were normalized using the *18S rRNA* level as the standard. The values shown in the bar graphs are related to an arbitrary unit of the HB2-C cells (set to 1), and the means ± SE values (*n* = 3) are given: ^a^ *p* < 0.05 vs. HB2-C cells; ^b^ *p* < 0.05 vs. HB2-L3bp cells; ^c^ *p* < 0.05 vs. HB2-L3-L3bp cells; and ^d^ *p* < 0.05 vs. HB2-L3 cells. (**B**) Expression levels of Flag-Lgals3 and Flag-Lgals3bp proteins in the cell lysate and the culture medium of HB2 transfectants. Cellular protein extracts and cell culture media of each HB2 transfectant at different differentiation stages (N: not differentiated; ED: early phases of brown adipocyte differentiation; MB: mature brown adipocyte) were collected and then subjected to Western blot analysis. Flag-tag proteins were detected by anti-DYKDDDDK (Flag) antibody. β-tubulin probe was used as a loading control. (**C**) Relative expression levels of Flag-Lgals3 or -Lgals3bp protein in culture medium of HB2-L3 or -L3bp cells. The values shown in the bar graphs are related to an arbitrary unit of the N stage (set to 1), and the means ± SE values (*n* = 3) are given: ^a^ *p* < 0.05 vs. N stage; ^b^ *p* < 0.05 vs. ED stage. (**D**) Flag-tag protein in mature HB2-C, HB2-L3, or HB2-L3bp cells was stained using an anti-Flag antibody. Representative photomicrographs of each cell are shown. Each scale bar indicates 50 μm.

Enhanced expression of *Lgals3* gene is found in the WAT and the BAT of obese mice, and the insulin signal was reported to be altered in the WAT of Lgals3 knock out (KO) mice, but the influence of Lgals3 on brown adipocytes remains unknown [41,42]. Lgals3bp was identified as a protein that binds to Lgals3 and Lgals1, but its effect on brown adipocytes is also unknown [43]. As shown in Table 3, the expressions of genes for *Lgals3* and *Lgals3bp* are thought to be greatly influenced by HFD intake and PA. Therefore, we attempted to establish an overexpression of Flag-tagged Lgals3 and Lgals3bp in HB2 cells. Real-time PCR analysis showed a significant increase in the expressions of *Lgals3* and *Lgals3bp* mRNAs in Flag-tagged Lgals3 (HB2-L3 cells), Lgals3bp (HB2-L3bp cells), or both (HB2-L3-L3bp cells) when compared with that in control cells (HB2-C cells) (Figure 1A). Moreover, expression of exogenous Flag-tagged proteins in cell lysate and Flag-Lgals3 or Lgals3bp protein secretion into cell culture medium were confirmed in HB2-L3, -L3bp, and -L3-L3bp cells (Figure 1B). As shown in Figure 1B,C, in the differentiation process of HB2 cells into brown adipocytes, Flag-tagged Lgals3 protein was secreted the most during the early stages of differentiation, while the secretion from mature brown adipocytes was decreased by comparison. In contrast, the secretion of the Flag-tagged Lgals3bp protein from mature brown adipocytes was the most prominent. In addition, although the Flag-tagged Lgals3 protein was secreted at each stage of brown adipocyte differentiation, most of this protein was uniformly present in the cytoplasm (Figure 1D). Furthermore, the expression of Flag-tagged Lgals3bp in the cytoplasm was heterogeneous compared with that of the Flag-tagged Lgals3 protein (Figure 1D).

### 2.6. Effects of Lgals3 and Lgals3bp on Brown Adipocyte Differentiation

**Figure 2:**
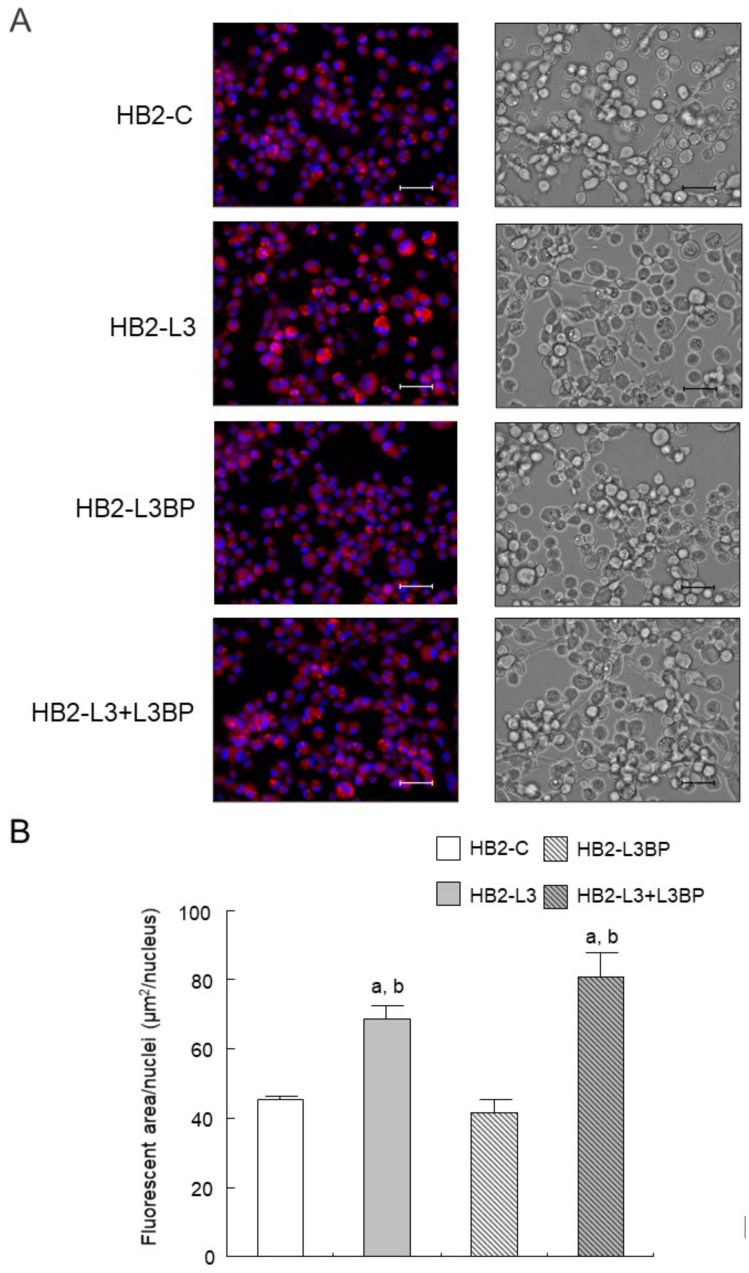
Effects of Lgals3 and Lgals3bp on lipogenesis in HB2 cells. (**A**) Lipid droplets in HB2 transfectants after mature differentiation were stained red using a Nile red solution. Representative photomicrographs (left) and corresponding bright field images (right) are shown. Each scale bar indicates 50 μm. (**B**) Measured areas of staining with Nile red per the number of Hoechst positive nuclei are calculated in three different fields of view. The mean ± SE values (*n* = 3) are given: ^a^ *p* < 0.05 vs. HB2-C cells; and ^b^ *p* < 0.05 vs. HB2-L3bp cells.

**Figure 3:**
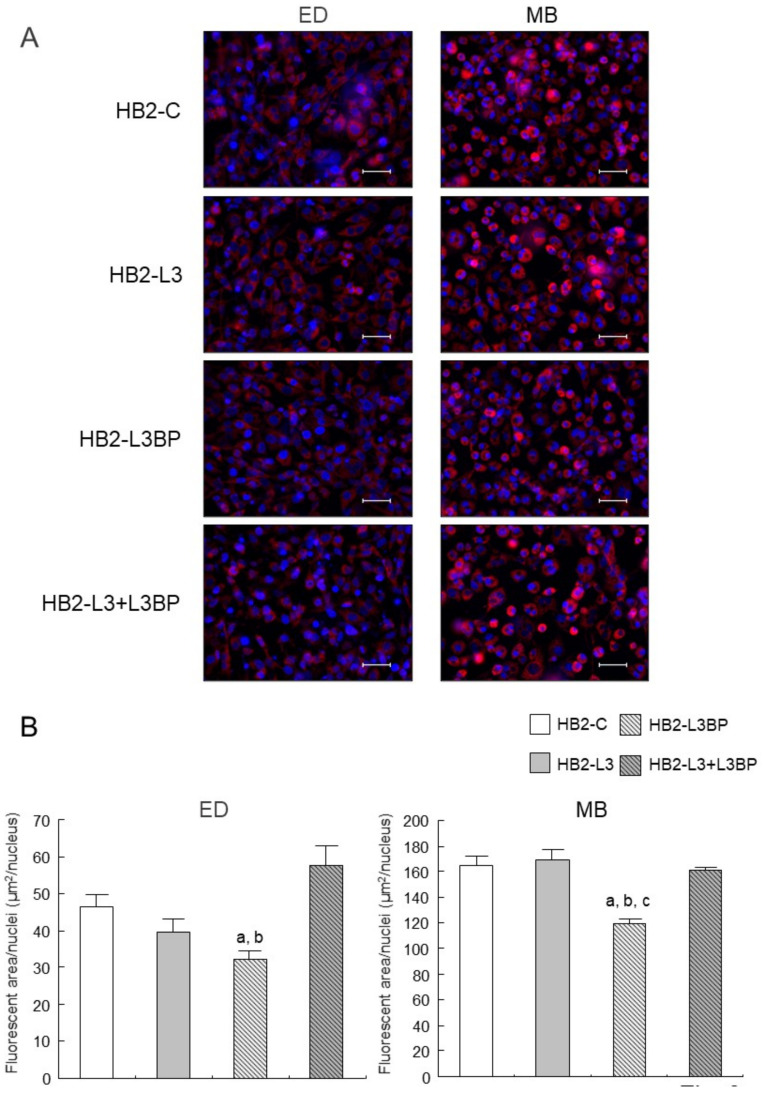

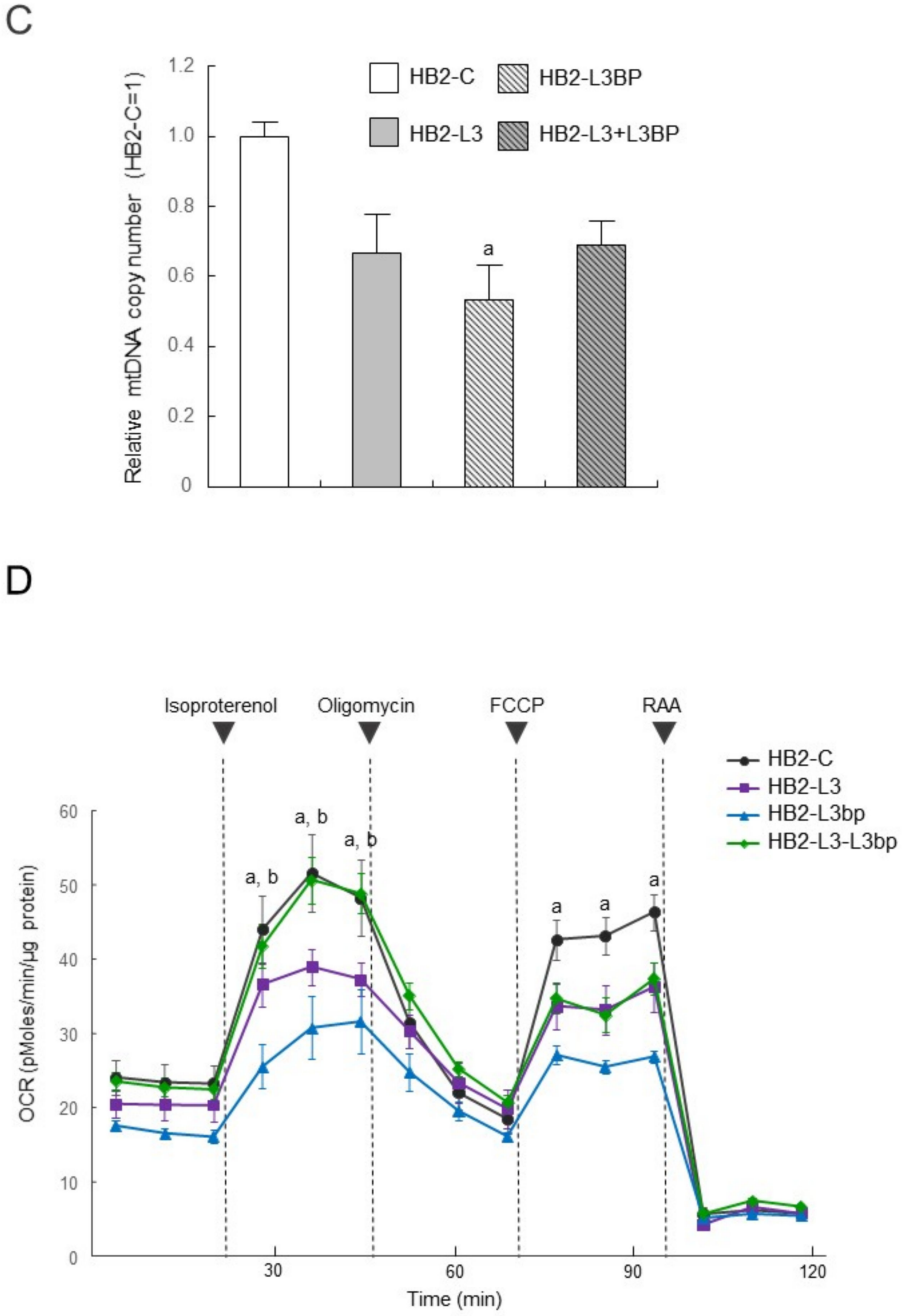
Effects of Lgals3 and Lgals3bp on the mitochondrial content in HB2 cells. (**A**) Intracellular mitochondria in HB2 transfectants during or after differentiation were stained red using a MitoTracker solution. Representative photomicrographs are shown. Each scale bar indicates 50 μm. ED: early phases of brown adipocyte differentiation; MB: mature brown adipocyte. (**B**) Measured areas of staining using a MitoTracker per the number of Hoechst positive nuclei are calculated in three different fields of view. The mean ± SE values (*n* = 3) are given: ^a^ *p* < 0.05 vs. HB2-C cells; ^b^ *p* < 0.05 vs. HB2-L3-L3bp cells; and ^c^ *p* < 0.05 vs. HB2-L3 cells. (**C**) The mtDNA copy number in mature HB2 transfectants. The mtDNA was amplified using the PCR protocol with primers that specifically amplify mouse mtDNA. The values shown in the bar graphs are related to an arbitrary unit of the HB2-C cells (set to 1), and the means ± SE values (*n* = 3) are given: ^a^ *p* < 0.05 vs. HB2-C cells. (**D**) OCR in mature HB2 transfectants. OCRs were measured with the sequential injection of isoproterenol (1 µm), oligomycin (5 µm), FCCP (10 µM), rotenone (R; 0.5 µm), and antimycin A (AA: 0.5 µm) at the indicated times. The means ± SE values (*n* = 5) are given: ^a^ *p* < 0.05, HB2-L3bp cells vs. HB2-C cells; ^b^ *p* < 0.05, HB2-L3bp cells vs. HB2-L3-L3bp cells.

Intracellular lipid droplet formation after mature brown adipocyte differentiation of HB2-L3 and HB2-L3-L3bp cells was significantly enhanced compared with that of HB2-C and HB2-L3bp cells (Figure 2). On the other hand, the amounts of intracellular mitochondria in HB2-L3bp cells were significantly decreased compared with those of HB2-C and HB2-L3 cells in the early differentiation stage and also by comparison with all other cell groups after mature brown adipocyte differentiation (Figure 3A,B). Furthermore, the copy number of mitochondrial DNA (mtDNA) in each mature HB2 transfectant was examined using primers that were reported to specifically amplify mouse mtDNA [44]. The results showed that the copy number of mtDNA in HB2-L3bp cells was significantly lower than that in HB2-C cells (Figure 3C). In addition, when we examined mitochondrial function by measuring the oxygen consumption rate (OCR), we found that the OCR of HB2-L3bp was significantly suppressed when stimulated by isoproterenol compared with that of either HB2-C or HB2-L3-L3bp cells, which was also the case when stimulated with the potent uncoupler carbonyl cyanide-p-trifluoro-methoxyphenylhydrazone (FCCP) compared with that of HB2-C cells (Figure 3D).

**Figure 4:**
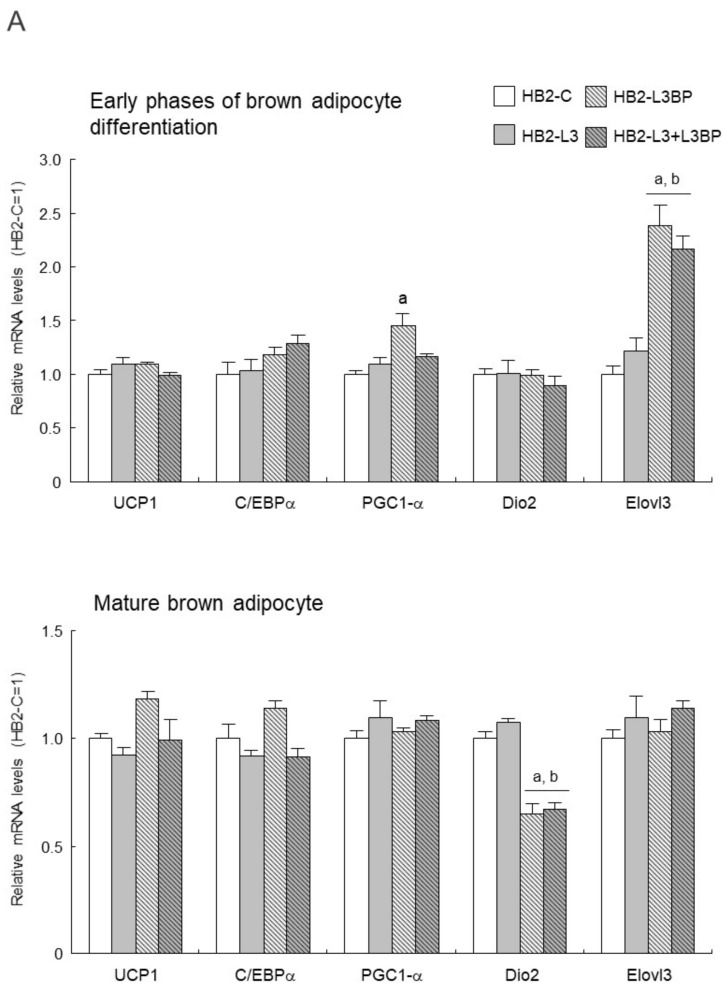

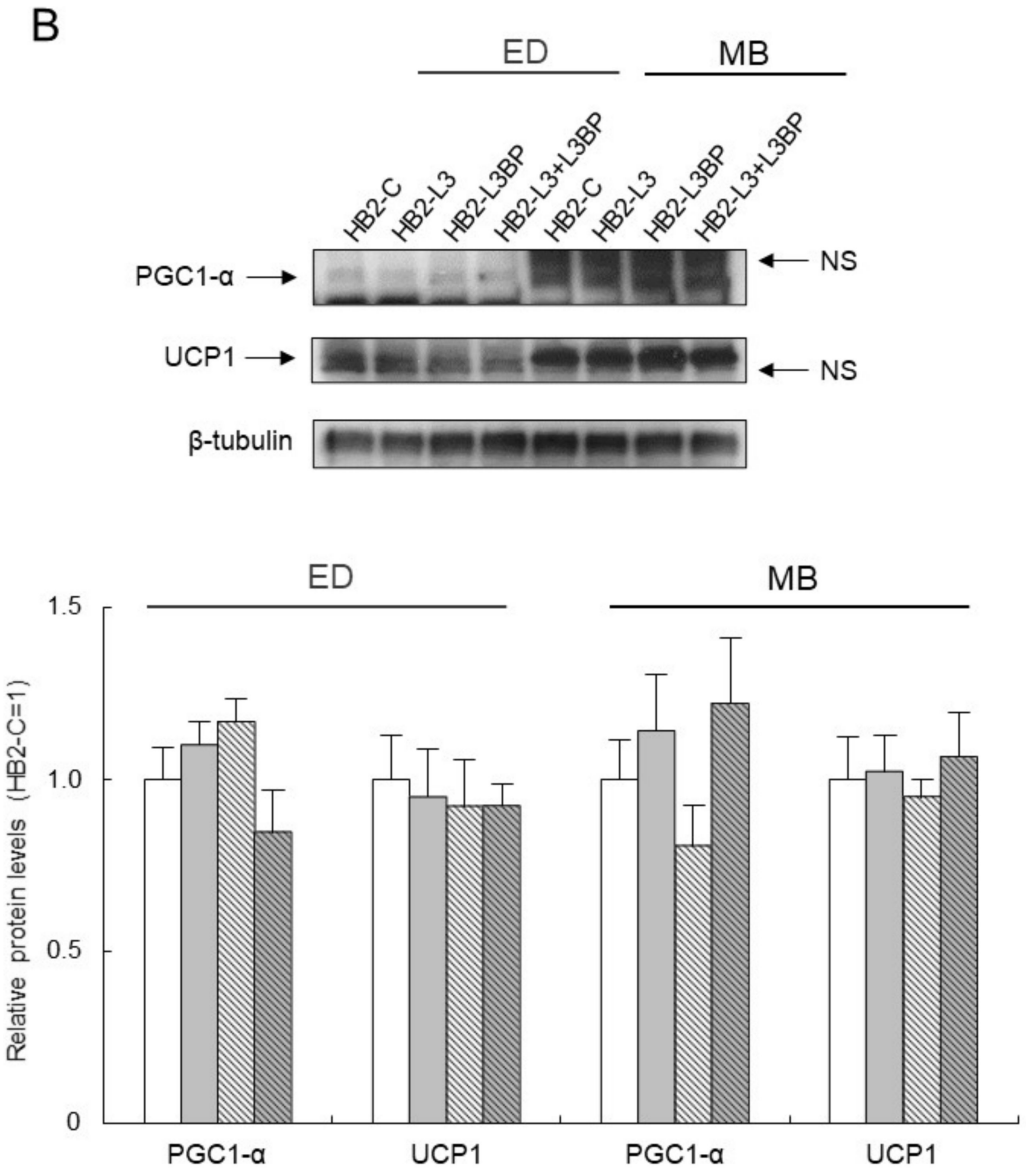
Effect of Lgals3 and Lgals3bp on the expression of brown adipocyte differentiation-related factors. (**A**) Expression levels of brown adipocyte differentiation-related mRNAs in HB2 transfectants. Total RNAs were extracted during or after differentiation from each HB2 transfectant and then subjected to real time quantitative PCR. The expression level of each mRNA was normalized using the *18S rRNA* level as the standard. The values shown in the bar graphs are related to an arbitrary unit of the HB2-C cells (set to 1), and the means ± SE values (*n* = 3) are given: ^a^ *p* < 0.05 vs. HB2-C cells; and ^b^ *p* < 0.05 vs. HB2-L3 cells. (**B**) Expression levels of PGC1-αand UCP1 protein in HB2 transfectants. Representative data from western blot analyses are shown in the superior region. Expression levels of PGC1-α or UCP1 proteins were normalized to those of the expression levels of β-tubulin. The data are expressed as ratios with the value of the HB2-C cells = 1. The mean ± SE (*n* = 3) is given. ED: early phases of brown adipocyte differentiation; MB: mature brown adipocyte; NS: nonspecific band.

As for the expression levels of mRNA for brown adipocyte differentiation-related factors in HB2 transfectants (Figure 4A), in the early stages of brown adipocyte differentiation, expression of the *PGC1-α* gene was significantly upregulated in HB2-L3bp cells compared with that of HB2-C cells. Moreover, *Elovl3* gene expression was significantly increased in the Lgals3bp-transfected cells. After differentiation of the mature brown adipocytes, the expression of the *Dio2* gene was significantly decreased by the introduction of Lgals3bp into HB2 cells. No significant effect of Lgals3 or Lgals3bp overexpression was observed in the expression of other genes such as *UCP1* (Figure 4A). On the other hand, the protein expression of PGC-1α and UCP1 in the early phases of brown adipocyte differentiation and after maturation was not significantly different among the HB2 transfectants (Figure 4B).

## 3. Discussion

In the present study, we identified humoral factors from HB2 brown adipocytes similar to those reported by Villarroya et al. [30] using murine brown preadipocytes in BAT. Among these humoral factors, Ccl9, Lgals3, and Lgals3bp were found to be brown adipokines with gene expressions that were largely influenced by obesity and PA. Ccl9, which is also known as macrophage inflammatory protein 1-γ, is a chemokine belonging to the CC chemokine family [45] and is known to play an important role in anti-leukemia and bone resorption procedures. Although there are very few reports of the effect of Ccl9 on adipocytes, it is known to inhibit the differentiation of white adipocytes [46].

The Lgals3 protein (galectin-3) is involved in biological processes such as cell adhesion, inflammation, and apoptosis. Lgals3 is upregulated in the WAT and the BAT of obese mice and can attenuate insulin signaling in white adipocytes [41]. Furthermore, obese and diabetic individuals were shown to have higher blood levels of the Lgals3 protein, which parallels the deterioration of glucose homeostasis and suggests that Lgals3 may be involved in the development of obesity and type 2 diabetes [41]. In addition, studies using Lgals3 KO mice reported decreases in body and fat masses in HFD-fed Lgals3 KO mice by comparison with control mice [47]. However, another study using Lgals3 KO mice demonstrated that Lgals3 deficiency accelerates adiposity, levels of adipose tissue, and systemic inflammation associated with altered glucose homeostasis [48,49]. Additionally, Lgals3 is known to stimulate the differentiation of preadipocytes into mature white adipocytes in vitro [47]. On the other hand, human Lgals3bp has long been regarded as an important clinical tumor biomarker associated with disease diagnosis, negative prognosis, and poor response to therapy [50]. Lgals3bp is also secreted by human visceral WAT and inhibits white adipocyte differentiation [51]. Lgals3 and Lgals3bp proteins are secreted by macrophages and are involved in immune responses. Inflammatory stimuli to macrophages promote the secretion of Lgals3 protein, which is known to be involved in infection and inflammation as an immune regulator with functions such as regulating leukocyte function as well as inducing angiogenesis in cancer cells and regulating apoptosis in tumor cells [52]. Lgals3 protein is also related to the onset of tissue fibrosis [53]. On the other hand, Lgals3bp is abundantly expressed in human M1 macrophages and is suggested to be a macrophage inflammatory marker in carotid artery disease in women with human immunodeficiency virus or hepatitis C virus infections [54]. Results of the present study suggest that inflammatory changes due to macrophage infiltration may be occurring in the BAT of obese mice. In fact, Krautbauer et al. [42] found that the expression of pro-inflammatory cytokine and macrophage marker genes was upregulated in the BAT of HFD-induced obese mice and genetically ob/ob mice. Therefore, the gene expressions of *Lgals3* and *Lgals3bp* in macrophages infiltrating BAT may be partially responsible for the elevated expressions of those genes in the BAT of obese mice. In addition, the enhanced expressions of fibronectin and collagen genes, which play important roles in tissue fibrosis, were also observed in the BAT of obese mice. These observations suggest the possibility that Lgals3 brings about increases in fibrosis-related factors in the BAT of obese subjects.

Overexpression of Lgals3 in HB2 cells resulted in enhanced intracellular lipogenesis, suggesting that Lgals3 may play an important role in the differentiation process of brown adipocytes, particularly in lipogenesis. On the other hand, the mitochondrial content of HB2-L3bp cells was reduced compared with control cells in the early phases of brown adipocyte differentiation and after maturation. Furthermore, after the differentiation of mature brown adipocytes, the copy numbers of mtDNA and OCR were significantly lower than those of HB2-C cells when stimulated with isoproterenol or the potent uncoupler FCCP. Therefore, Lgals3bp is suggested to suppress mitochondrial biogenesis. On the other hand, the expression levels of *Elovl3* genes were significantly increased in HB2-L3bp cells in the early stages of differentiation compared with control cells, and the expression levels of PGC-1α proteins in HB2-L3 cells during brown adipocyte differentiation did not differ significantly from the other three HB2 transfectants. Nonetheless, mitochondrial content was decreased. The reason for this is unknown, but the expressions of PGC-1α and Elovl3 did not seem to affect the mitochondrial biogenesis of HB2-L3bp cells. The *Dio2* gene encodes type II deiodinase, which converts thyroxine (T_4_) to triiodothyronine (T_3_). In particular, in skeletal muscles and BAT, the converted T_3_ stimulates protein synthesis relating to mitochondrial biogenesis, which results in higher energy expenditure [36]. Moreover, when Christoffolete et al. [55] generated Dio2 KO mice and examined their BAT, they showed a cold-induced overexpression of the *UCP1* gene in their BAT, but they did not exhibit increases in BAT lipogenesis or adaptive thermogenesis. In the present study, we found that Lgals3bp decreased the expression of the *Dio2* gene in mature brown adipocytes. Therefore, it is possible that the reduced expression of *Dio2* gene is involved in the reduced mitochondrial content that was observed in the HB2-L3bp cells. Nevertheless, although *Dio2* gene expression was decreased in mature HB2-L3-L3bp cells, the mitochondrial content was not significantly different from that of control cells. Thus, the repressive effect of mitochondrial biogenesis with Lgals3bp was not simply due to the decreased expression of *Dio2* gene but also to other causes. In this study, we mainly observed the mRNA expression levels of brown adipocyte differentiation markers. In order to elucidate the molecular mechanism of the effect of Lgals3bp on mitochondrial biogenesis, it will be necessary to further investigate not only mRNA expression levels but also changes in related protein modifications.

As described above, Lgals3 was expected to enhance lipogenesis in brown adipocytes, while Lgals3bp was expected to inhibit mitochondrial biogenesis by stimulating differentiation. However, in the present study, although Lgals3bp proteins were identified as a factor in binding to Lgals3 protein and enhancing Lgals3 protein activity [37], Lgals3 and Lgals3bp did not appear to work in concert. This observation could be attributed to the fact that simultaneous infection of HB2 cells by Lgals3 and Lgals3bp via lentiviruses reduces gene transfer to about half that of a single infection. However, the Flag-Lgals3 protein is mainly found in the cytoplasm, and its secretion appears to be lower than that of the Flag-Lgals3bp protein. It is likely that each protein functions independently due to its different localization. Nevertheless, since it cannot be denied that Lgals3 and Lgals3bp bind and exert cooperative effects on brown adipocytes, further investigation is needed.

It is well known that PA and exercise are recognized as effective means of preventing and improving obesity and lifestyle-related diseases. The purpose of our investigation was to examine the effect of PA on obesity-induced remodeling in BAT and to find novel effects of PA on the prevention and the improvement of obesity and lifestyle-related diseases. Therefore, we did not establish a group that exclusively engaged in PA.

A wide variety of adipokines secreted from WAT have been identified, and it has become clear that dysregulated expression of adipokines due to obesity is closely related to the onset of lifestyle-related diseases [9,10,11]. The effects of PA and exercise on adipokine expression in WAT were reported in many studies [12,13]. The influence of PA and exercise on adipokine expression in WAT and in blood levels has not always been consistent among reports due to differences in experimental subjects, exercise intensity, and duration [12,13]. However, PA and exercise is believed to have a positive effect. With respect to BAT, the present study revealed that obesity causes an abnormal expression of several fluid factors, presumably brown adipokines, and a decrease in the expression of genes for brown adipocyte differentiation markers such as *PGC1-α*. Therefore, it is inferred that obesity causes dysfunction in BAT as well as WAT obesity. Physical activity seems to partially suppress the dysfunction of BAT caused by obesity. Nevertheless, there is disagreement regarding the effects of exercise on the functions of BAT. For example, in an 8 week study of running exercise in rats using treadmills, decreases in BAT mass and UCP1 expression were observed [56]. By contrast, swimming exercise at a water temperature that does not cause heat production by shivering enhances the function of BAT [57]. Furthermore, several studies demonstrated increased thermogenic and metabolic activities of BAT and UCP1 expression after exercise in rodents [58]. Thus, further studies including the examination of brown adipokines are needed to clarify the effects of PA and exercise on BAT.

In the present study, we observed the dysregulated expressions of many genes, which included brown adipokines and differentiation-related factors, in the BAT of obese mice subjected to a HFD intake. PA attenuated those abnormal gene expressions, albeit only partially. Specifically, the expressions of genes for putative brown adipokines such as *Ccl9*, *Lgals3*, and *Lgals3bp* were substantially upregulated by obesity, and PA reduced their expressions. In brown adipocytes, Lgals3 caused increased lipogenesis, and Lgals3bp induced decreases in the mitochondrial content. However, overexpression of Lgals3 or Lgals3bp in HB2 cells did not correspond to BAT in mice subjected to a HFD intake and PA. These results suggest that Lgals3 or Lgals3bp may exert their functions in brown adipocytes through functions such as protein modification instead of the regulation of gene expression. Further investigations into the effects of Lgals3 and Lgals3bp on BAT are necessary, and these include elucidation of the molecular mechanisms as well as in vivo studies.

## 4. Methods

### 4.1. Cell Culture

The brown preadipocytes of HB2 mice were kindly provided by Professor M. Saito (Tenshi University, Sapporo, Japan) [29] and were maintained in Dulbecco’s modified eagle medium (DMEM) (Sigma Chemical, St. Louis, MO, USA) supplemented with 10% fetal calf serum (FCS). Differentiation to brown adipocytes was induced by treatment with 1 μM dexamethasone (Sigma Chemical) and 0.5 mM 3-isobutyl-1-methylxanthine (Wako, Osaka, Japan) for 48 h (early differentiation phase). The treated cells were maintained in DMEM containing 10 μg/mL of insulin (Wako) and T_3_ (Wako) for 96 h to accumulate triglycerides (inducing mature brown adipocytes). Fully differentiated HB2 cells were incubated with phenol red and FCS-free DMEM supplemented with Insulin-Transferrin-Selenium (Thermo Fisher Scientific, Waltham, MA, USA) for 24 h. Incubated medium was concentrated using Amicon Ultra (Merck Millipore, Darmstadt, Germany), and protein identification in the concentrated medium was performed via mass spectrometry.

### 4.2. Proteomic Analysis for the Identification of Fluid Factors from Brown Adipocytes

Proteomic analyses were performed as described previously [59]. All of the fractionated peptides described above were injected into a trap column (C18, 0.3 × 5 mm; L-column, Chemicals Evaluation and Research Institute, Tokyo, Japan) and an analytical column (C18, 0.075 × 120 mm; Nikkyo Technos, Tokyo, Japan), and both were attached to a nano liquid chromatography-tandem mass spectrometry (nanoLCMS/MS) system. The nanoLC-MS/MS analysis was conducted using an LTQ Orbitrap Velos mass spectrometer (Thermo Fisher Scientific) equipped with a nanoLC interface (KYA, Tokyo, Japan) and a nano high-performance liquid chromatography (nanoHPLC) system (DiNa; KYA). Purified peptides from the nanoLC were introduced into the LTQ Orbitrap Velos, a hybrid ion-trap Fourier transform mass spectrometer. Full MS and MS/MS scans were followed by higher energy collisionally activated dissociation. The database search engines Proteome Discoverer 1.4 (Thermo Scientific) and MASCOT 2.6 (Matrix Science, Tokyo, Japan) were used to identify and quantify proteins from MS, MS/MS, and reporter ion spectra of peptides. Peptide mass data were matched by searching the murine protein database (UniprotKB/Swiss-prot). The false discovery rate (FDR) [60] was calculated via peptide sequence analysis using a Percolator [61]. High-confidence peptide identifications were obtained by setting a target FDR threshold of ≤1.0% at the peptide level. The numbers of identified peptide spectra matches (PSM) appear in Table 1.

### 4.3. Animal Care

Six-week-old male C57BL/6J mice (Sankyo Labo Service Corporation, Tokyo, Japan) were housed in cages in a temperature-controlled environment at 23 °C under a 12:12 h light–dark cycle. The mice were divided randomly into three groups (*n* = 4 each): (i) C mice—control mice fed normal chow for four months; (ii) HFD mice—mice given an HFD for four months; and, (iii) HFD + PA mice—PA mice given an HFD for four months. The HFD consisted of 60.7% (calorie ratio) fat, 17.9% protein, and 21.4% nitrogen-free extract (HFD-60; Oriental Yeast Co., ltd., Tokyo, Japan). HFD + PA mice were subjected to wheel running. The wheels were equipped with a permanent magnetic switch that activated a digital counter of wheel revolutions (Melquest Ltd., Toyama, Japan). Total wheel revolutions were recorded daily, and the total distance run per day was determined by multiplying the number of wheel rotations by wheel circumference. All experiments conducted in the present study were approved by the Experimental Animal Ethics Committee of the Kyorin University School of Medicine, Mitaka.

### 4.4. RNA Isolation and DNA Array Analysis

Total RNAs in the interscapular BAT of mice or HB2 brown adipocytes were prepared by using Isogen (Nippon Gene, Toyama, Japan). The concentration and the purity of extracted total RNA of BAT were measured using the NanoDrop system (Thermo Fisher Scientific, Waltham, MA, USA). In addition, the RNA integrity number (RIN) was measured using an Agilent 2200 TapeStation (Agilent Technologies, Santa Clara, CA, USA) to confirm the quality of extracted RNA. cDNA synthesis via purified total RNA, fragmentation, and labeling was performed using a GeneChip WT PLUS Reagent Kit (Thermo Fisher Scientific) according to the manufacturer’s instructions. Subsequently, the gene expression profiles of each group were determined using Clarion S arrays for mice (Thermo Fisher Scientific).

### 4.5. Real Time Quantitative PCR

First-strand cDNA of HB2 brown adipocytes was obtained by incubating total RNA samples (2 μg) with reverse transcriptase (PrimeScrip II 1st strand cDNA Synthesis Kit, Takara Bio, Kusatsu, Japan) in a reaction mixture (20 μL). The RT product was amplified using a KAPA SYBR Fast qPCR Kit (Kapa Biosystems, Wilmington, MA, USA) in triplicate in a 7500 Real Time PCR System (Applied Biosystems). PCR conditions were 50 °C for 2 min and 95 °C for 10 min followed by 45 cycles of 95 °C for 15 s and 60 °C for 1 min. An 18s rRNA gene was used as an internal control, and the primer sequences were referenced from previous studies. Quantitative real-time PCR was performed for brown adipocyte marker genes such as 18S rRNA, Lgals3, Lgals3bp, UCP1, PGC-1α, Dio2, Elovl3, and C/EBPα using their specific primers. Each primer sequence was referenced in previous studies [28,43,62,63]. The 18S rRNA was used as an internal control.

The mtDNA in fully differentiated HB2 transfectants was extracted using an mtDNA Extractor CT kit (Wako) according to the manufacturer’s instructions. The extracted mtDNA was amplified using the PCR protocol described above with primer sets that were reported to specifically amplify mouse mitochondrial DNA [44]. Each value was corrected for the corresponding nDNA (β2- macroglobulin DNA) value.

### 4.6. Lentiviral Vector Construction and Lentivirus Production

Full-length mouse Lgals3 and Lgals3bp cDNA with a FLAG sequence at the 3-terminus was subcloned into the pLVSIN-EF1α vector (Takara Bio) at HindIII and XhoI sites. The lentiviral expression vectors of Lgals3, Lgals3bp, and a mock version were produced using the Lentiviral High Titer Packaging Mix (Takara Bio) and Lenti-X 293T cells (Takara Bio) according to the manufacturer’s recommendations. A cell culture medium containing each lentivirus was concentrated using a Lenti-X™ Concentrator (Takara Bio), and the lentiviral titer was measured using Lenti-X™ GoStix™ Plus (Takara Bio). Almost 1 × 10^6^ of IFU/mL lentivirus was infected with HB2 cells. Infected cells were selected in medium containing 1mg/mL of G418 (Geneticin; Wako).

### 4.7. Western Blot Analysis

Cell culture medium and lysate from lentivirus-infected HB2 cells was subjected to 10% TGX FastCast gel (Bio-Rad Laboratories, Hercules, CA, USA) electrophoresis and transferred onto a nylon membrane. The membrane was blocked using TBS T (20 mmol/L of Tris HCl, pH 7.5, 137 mM of NaCl, and 0.1% Tween 20) containing 5% nonfat dry milk and was then probed with an anti DYKDDDDK (Flag) tag (WAKO), PGC1-α (Santa Cruz Biotechnology, Santa Cruz, CA, USA), UCP1 (Abclonal, Tokyo, Japan), or β-tubulin (WAKO) antibody. After washing with TBS T, the bound antibody was detected using an ECL system (Amersham, Buckinghamshire, UK).

### 4.8. Cell Staining

After the induction of differentiation into brown adipocytes, HB2 cells infected with lentivirus were washed with phosphate buffer saline (PBS) and then fixed with 3.7% paraformaldehyde in PBS for 15 min. After fixation, these cells were permeabilized with 0.1% Triton X-100 in PBS containing 5% normal goat serum (NGS-PBSTX). The cells were then incubated with 5 µg/mL of the anti- DYKDDDDK (Flag)-tag antibody in NGS-PBSTX for 1.5 h at room temperature. After this incubation, the cells were washed and incubated for another 1 h with the Alexa Flour 488-conjugated anti-mouse IgG antibody (Molecular Probes, Thermo Fisher Scientific). The stained cells were observed with a fluorescent microscope (model BZ-X700, Keyence corporation, Osaka, Japan).

The intracellular lipid accumulations and mitochondria in HB2 cells were analyzed by Nile red (Wako) and MitoTracker (Thermo Fisher Scientific) staining, respectively. HB2 cells were washed twice with PBS and then were stained either with Nile red (500 nM) for 15 min at room temperature or with MitoTracker (200 nM) for 60 min at 37 °C and washed twice with PBS. After Nile red or MitoTracker staining, the nuclei in these cells were stained with Hoechst 33342 (Thermo Fisher Scientific). The stained cells were observed under a fluorescent microscope, and the light-emitting area was then measured using a BZ-X Analyzer (Keyence corporation). Moreover, we chose three fields of view for each of the groups stained with either Nail red or MitoTracker and calculated the light-emitting area per number of nuclei.

### 4.9. Measurement of OCR

Fully differentiated cells HB2-C, -L3, L3bp, and -L3-L3bp were seeded into collagen I coated (KOKEN, Tokyo, Japan) XFe24 Microplates (Seahorse Bioscience, North Billerica, MA, USA) at a density of 50,000 cells/well and were incubated in a CO2 incubator for 24 h, and then cells were washed three times and incubated in Seahorse XF DMEM Medium, pH 7.4 (Seahorse Bioscience) supplemented with 25 mM glucose and 1 mM sodium pyruvate for 1 h. The OCR was measured using a Seahorse XFe24 flux analyzer (Seahorse Bioscience). The compounds used to determine OCR included 1 μM isoproterenol (Sigma), 5 μM oligomycin (Sigma), 10 μM FCCP, 0.5 μM antimycin A (Sigma), and 0.5 μM rotenone (Sigma). OCRs were normalized to the total protein content.

### 4.10. Statistical Analysis

Values are represented as the means ± standard error (SE). The significance of multiple comparisons was determined using one-way analysis of variance (ANOVA) followed by a Bonferroni’s post hoc test. *p* < 0.05 was considered statistically significant.

The intensities of bands from the Western blot analyses were quantified using the National Institute of Health Image software.

## Figures and Tables

**Table 1:** List of identified humoral factors secreted from mature HB2 brown adipocytes.

Accession	Protein	PSMs
P01027	Complement C3	629
P04117	Fatty acid-binding protein, Adipocyte (Fabp4)	337
Q61646	Haptoglobin	194
Q921I1	Serotransferrin	130
P11152	Lipoprotein lipase	107
P16045	Galectin-1	72
Q61207	Sulfated glycoprotein 1	62
Q99P87	Resistin	62
P08226	Apolipoprotein E	56
Q05816	Fatty acid-binding protein, epidermal	55
P10605	Cathepsin B	38
P03953	Complement factor D	37
P97298	Pigment epithelium-derivedf actor	35
Q8BPB5	EGF-containing fibulin-likeextracellular matrix protein 1	25
Q07797	Galectin-3-binding protein	23
Q62356	Follistatin-related protein 1	22
P10148	C-C motif chemokine 2	11
P16110	Galectin-3	9
P51670	C-C motif chemokine 9	6
Q02788	Collagen alpha-2(VI) chain	144
Q04857	Collagen alpha-1(VI) chain	134
P11276	Fibronectin	120
Q01149	Collagen alpha-2(I) chain	103
P02469	Laminin subunit beta-1	85
P02468	Laminin subunit gamma-1	71
P97927	Laminin subunit alpha-4	64
P08121	Collagen alpha-1(III) chain	62
P11087	Collagen alpha-1(I) chain	44

**Table 2:** Levels of body mass, WAT and BAT mass of control, HFD, and HFD + PA mice.

	Control Mice	HFD Mice	HFD + PA Mice
Body mass (g)	26.90 ± 0.84	37.62 ± 1.62	33.78 ± 0.80
Epididymal WAT mass per body mass (%)	1.20± 0.10	5.59 ± 0.26 ^a^	4.40 ± 0.07 ^a,b^
Interscapular BAT mass per body mass (%)	0.24 ± 0.01	0.29 ± 0.05	0.23 ± 0.02

The mean ± SE value (*n* = 4) is given. ^a^ *p* < 0.05 vs. control mice. ^b^ *p* < 0.05 vs. HFD mice.

**Table 3:** Expression levels of mRNA for representative humoral factors in the interscapular BAT of control, HFD, and HFD + PA mice.

Gene Symbol	Gene Expression Level by DNA Array (log2)		
Fluid factors found to be secreted by HB2 brown adipocytes	Control mice	HFD mice	HFD + PA mice
C3	14.53 ± 0.09	13.12 ± 0.18 ^a^	13.07 ± 0.26 ^a^
Fabp4	19.86 ± 0.08	19.86 ± 0.07	19.86 ± 0.08
Lgals3	4.31 ± 0.27	11.54 ± 1.44 ^a^	7.72 ± 1.04 ^b^
Lgals3bp	9.05 ± 0.21	12.00 ± 0.68 ^a^	10.35 ± 0.24 ^b^
Cfd	18.24 ± 0.58	13.97 ± 0.65 ^a^	11.78 ± 0.40 ^a,b^
Ccl2	5.82 ± 0.50	8.13 ± 0.45 ^a^	6.45 ± 0.30 ^b^
Ccl9	8.09 ± 0.41	12.71 ± 1.43 ^a^	10.10 ± 0.85
Trf	12.67 ± 0.27	11.35 ± 0.50 ^a^	10.50 ± 0.64 ^a^
ApoE	18.42 ± 0.18	17.78 ± 0.25 ^a^	16.62 ± 0.60 ^a,b^
Efemp1	10.29 ± 0.37	10.29 ± 0.26	9.19 ± 0.22 ^a^
Retn	16.40 ± 2.35	9.06 ± 0.27 ^a^	8.60 ± 0.42 ^a^
Hp	16.69 ± 0.21	17.63 ± 0.30	17.47 ± 0.33
Lgals1	15.15 ± 0.44	15.69± 0.62	15.69 ± 0.50
Fabp5	9.08 ± 0.38	11.01 ± 0.96	10.16 ± 0.72
Ctsb	18.45 ± 0.39	19.01 ± 0.25	18.82 ± 0.39
Psap	17.51 ± 0.25	17.95 ± 0.12	17.79 ± 0.39
Serpinf1	7.84 ± 0.81	8.24 ± 0.83	8.06 ± 0.76
Fstl1	8.02 ± 0.07	7.85 ± 0.25	7.64 ± 0.69
Col1a1	7.39 ± 0.70	8.69 ± 0.67	8.06 ± 0.76
Col1a2	7.49 ± 0.74	9.94 ± 0.95	8.58 ± 0.23
Col3a1	9.22 ± 0.28	12.50 ± 0.58 ^a^	11.04 ± 0.59
Col6a1	6.91 ± 0.13	8.01 ± 0.31 ^a^	6.92 ± 0.27^b^
Col6a2	8.09 ± 0.29	8.77 ± 0.31	8.43 ± 0.28
FN1	6.53 ± 0.30	8.70 ± 0.54 ^a^	7.48 ± 0.21
Lama4	11.82 ± 0.29	11.83 ± 0.26	11.60 ± 0.22
Lamb1	10.82 ± 0.29	9.21 ± 0.25 ^a^	9.44 ± 0.34 ^a^
Lamc1	7.76 ± 0.30	8.56 ± 0.31	8.49 ± 0.25
MMP12	4.37 ± 0.70	12.73 ± 0.75 ^a^	10.34 ± 0.28 ^a,b^

The mean ± SE value (*n* = 4) is given. ^a^ *p* < 0.05 vs. control mice. ^b^ *p* < 0.05 vs. HFD mice.

**Table 4:** Expression levels of mRNA for macrophage markers and brown adipocyte differentiation-related factors in the interscapular BAT of control, HFD, and HFD + PA mice.

Gene Symbol	Gene Expression Level by DNA Array (log2)		
Macrophage markers	Control Mice	HFD mice	HFD + PA mice
Adgre1 (F4/80)	6.66 ± 0.20	9.29 ± 0.96 ^a^	7.96 ± 0.50
Mpeg1	5.81 ± 0.14	11.72 ± 0.54 ^a^	9.38 ± 0.78 ^a,b^
Itgam (CD11b)	5.51 ± 0.37	8.9 ± 0.77 ^a^	6.51 ± 0.49 ^a,b^
CD53	6.67 ± 0.20	11.04 ± 0.83 ^a^	8.74 ± 0.47
CD68	8.09 ± 0.29	13.55 ± 1.24 ^a^	11.11 ± 0.59
Brown adipocyte differentiation-related factors	Control mice	HFD mice	HFD + PA mice
UCP1	19.85 ± 0.09	19.76 ± 0.06	19.74 ± 0.06
PGC1-α	14.50 ± 0.56	13.25 ± 0.20 ^a^	13.75 ± 0.31
Dio2	12.98 ± 0.37	10.37 ± 0.08 ^a^	10.02 ± 0.60 ^a^
Elovl3	14.10 ± 0.29	13.27 ± 0.46 ^a^	14.17 ± 0.52
LPL	18.07 ± 0.28	17.05 ± 0.09 ^a^	16.95 ± 0.12 ^a^
Cebpα	13.94 ± 0.22	13.35 ± 0.09	13.62 ± 0.14

The mean ± SE value (*n* = 4) is given. ^a^ *p* < 0.05 vs. control mice. ^b^ *p* < 0.05 vs. HFD mice.

## Data Availability

The data that support the findings of this study are available from the corresponding author upon reasonable request.

## Abbreviations

Adipsin	complement factor D
ApoE	apolipoprotein E
BAT	brown adipose tissue
C/EBPα	CCAAT/enhancer binding protein α
Ccl9	C-C motif chemokine 9
Dio2	type II iodothyronine deiodinase
Efemp1	EGF-containing fibulin-like extracellular matrix protein 1
Elovl3	elongation of very long chain fatty acid protein 3
Fabp4	fatty acid binding protein 4
FCCP	carbonyl cyanide-p-trifluoro-methoxyphenylhydrazone
HFD	high-fat diet
mtDNA	mitochondrial DNA
Lgals3	lectin, galactose-binding, soluble 3
Lgals3bp	Lgals3 binding protein
OCR	oxygen consumption rate
PA	Physical activity
PGC-1α	peroxisome proliferator-activated receptor γ coactivator-1α
PSM’s	peptide-spectrum matches
RAA	rotenone-antimycin A
UCP1	uncoupling protein 1
WAT	white adipose tissue
